# 3D bio-printed scaffold-free nerve constructs with human gingiva-derived mesenchymal stem cells promote rat facial nerve regeneration

**DOI:** 10.1038/s41598-018-24888-w

**Published:** 2018-04-26

**Authors:** Qunzhou Zhang, Phuong D. Nguyen, Shihong Shi, Justin C. Burrell, D. Kacy Cullen, Anh D. Le

**Affiliations:** 1 Department of Oral and Maxillofacial Surgery and Pharmacology, University of Pennsylvania School of Dental Medicine, 240 South 40th Street, Philadelphia, PA19104 USA; 2 Division of Plastic and Reconstructive Surgery, University of Pennsylvania Perelman School of Medicine and Children’s Hospital of Philadelphia, 3400 Civic Center Blvd, Philadelphia, PA 19104 USA; 3 Department of Neurosurgery, University of Pennsylvania Perelman School of Medicine, 3320 Smith Walk, Philadelphia, PA19104 USA; 4 Department of Oral & Maxillofacial Surgery, Penn Medicine Hospital of the University of Pennsylvania, Perelman Center for Advanced Medicine, 3400 Civic Center Blvd, Philadelphia, PA 19104 USA

## Abstract

Despite the promising neuro-regenerative capacities of stem cells, there is currently no licensed stem cell-based product in the repair and regeneration of peripheral nerve injuries. Here, we explored the potential use of human gingiva-derived mesenchymal stem cells (GMSCs) as the only cellular component in 3D bio-printed scaffold-free neural constructs that were transplantable to bridge facial nerve defects in rats. We showed that GMSCs have the propensity to aggregate into compact 3D-spheroids that could produce their own matrix. When cultured under either 2D- or 3D-collagen scaffolds, GMSC spheroids were found to be more capable of differentiating into both neuronal and Schwann-like cells than their adherent counterparts. Using a scaffold-free 3D bio-printer system, nerve constructs were printed from GMSC spheroids in the absence of exogenous scaffolds and allowed to mature in a bioreactor. *In vivo* transplantation of the GMSC-laden nerve constructs promoted regeneration and functional recovery when used to bridge segmental defects in rat facial nerves. Our findings suggest that GMSCs represent an easily accessible source of MSCs for 3D bio-printing of scaffold-free nervous tissue constructs with promising potential application for repair and regeneration of peripheral nerve defects.

## Introduction

Peripheral facial nerve injuries are commonly caused by trauma, surgical removal of benign or malignant head & neck tumors and petrous bone surgery. These facial nerve injuries may lead to dysfunction of facial muscles, impaired sensation and/or painful neuropathies^1^. Currently, the so-called “gold standard” for reconstruction of segmental defects in facial nerves has been the autologous nerve graft, which provides a Schwann cell-rich structure to guide axonal regeneration. However, the permanent donor site morbidity, limited availability of sacrificial donor nerves, additional surgery and/or prolonged surgical time, significantly limit the clinical application of this method^1,2^. In an attempt to compensate for the drawbacks of autologous nerve grafts, the combination of using state-of-the-art 3D biofabrication technology, stem cells from readily-accessible sources, and various types of biomaterials/scaffolds for fabricating tissue engineered nerve constructs is emerging as a novel approach to facilitate peripheral nerve regeneration^3^.

3D bio-printing is a fabrication technology that precisely dispenses cell-laden biomaterials for the construction of complex 3D functional tissues or artificial organs^4^. 3D bio-printing is emerging as a novel approach in tissue engineering and regenerative medicine (TE/RM) to meet the growing need for transplantable tissues and organs^4,5^. Scaffold-free approaches mimic the fundamental developmental processes through tissue self-assembly, whereby cell spheroids in close contact with each other can spontaneously fuse into larger tissue units^6–9^. When cultured under non-adhesive conditions, mesenchymal stem cells (MSCs) tend to aggregate to form 3D-spheroids and exhibit improved biological properties and remarkable regenerative capability^10^. The 3D-spheroids also allow incorporation of multiple types of cells and reorganization into specialized structures, thus representing attractive building blocks for bioengineering scaffold-free tissues^6^. Therefore, 3D bio-printing of scaffold-free nerve tissue by using spheroid MSCs as the only cellular component represents a novel paradigm in this field.

Numerous types of stem cells have been assessed for the potential use for tissue engineering of nerve tissues. These sources of stem cells include embryonic stem cells (ESCs), induced pluripotent stem cells (iPSCs), neural stem (NSC) or progenitor cells (NPCs), and different tissue–derived MSCs^1,3,11^. However, the use of ESCs encounters ethical barriers and risks of immunogenicity and teratocarcinoma formation, thus significantly impeding their clinical application^12^. Even though the use of iPSCs derived from somatic cells can bypass issues associated with immune rejection and ethical concerns, variations among different iPSC populations in term of their differentiation and expansion capabilities as well as tumorigenic potentials constitute the major hurdle for their specific application in tissue engineering and regenerative medicine^11^. MSCs have the capacity to differentiate into functional glial and neuronal cells, however, the induction efficiency is quite variable due to the heterogeneity with respect to species, age, tissues from which they are derived, and culture conditions^11,13^. NSCs or NPCs isolated from embryonic or adult central nervous system as well as Schwann cells have been shown to be a promising source of seed cells for bioengineering neural tissues; nevertheless, it remains a challenge to expand and propagate them in multiple passages to meet the adequate quantity required for clinical use^14^. Therefore, there is an urgent need to identify an alternative and readily accessible source of stem cells for the fabrication of bioengineered nerve tissues.

We have isolated a unique subpopulation of MSCs from human gingival tissue (referred to as GMSCs), which is of neural crest origin^15,16^. Our recent study has shown that human GMSCs have the propensity to be induced into neural progenitor-like cells (NPCs), which displayed better therapeutic effects on peripheral nerve regeneration than the parental GMSC counterparts^17^. Here, we demonstrated that GMSCs are inclined to form compact 3D-spheroids, which had a diameter ranging from 400~500 µm and could also produce their own matrix, thus applicable for 3D bio-printing. When cultured under either 2D- or 3D-collagen scaffold conditions, GMSC spheroids more readily differentiated into both neuronal and Schwann-like cells than their adherent counterparts. Meanwhile, we have shown the feasibility of 3D bio-printing scaffold-free nerve constructs by using GMSC spheroids, which were implantable and promoted the repair and regeneration of rat facial nerve defects. Thus, the current study has provided substantial evidence that GMSCs represent an easily accessible source of stem cells that can be used as the cellular components for 3D bio-printing scaffold-free nerve constructs to meet the increasing clinical demand for peripheral nerve repair and regeneration.

## Results

### Generation of GMSC spheroids applicable for 3D bio-printing

**Figure 1:**
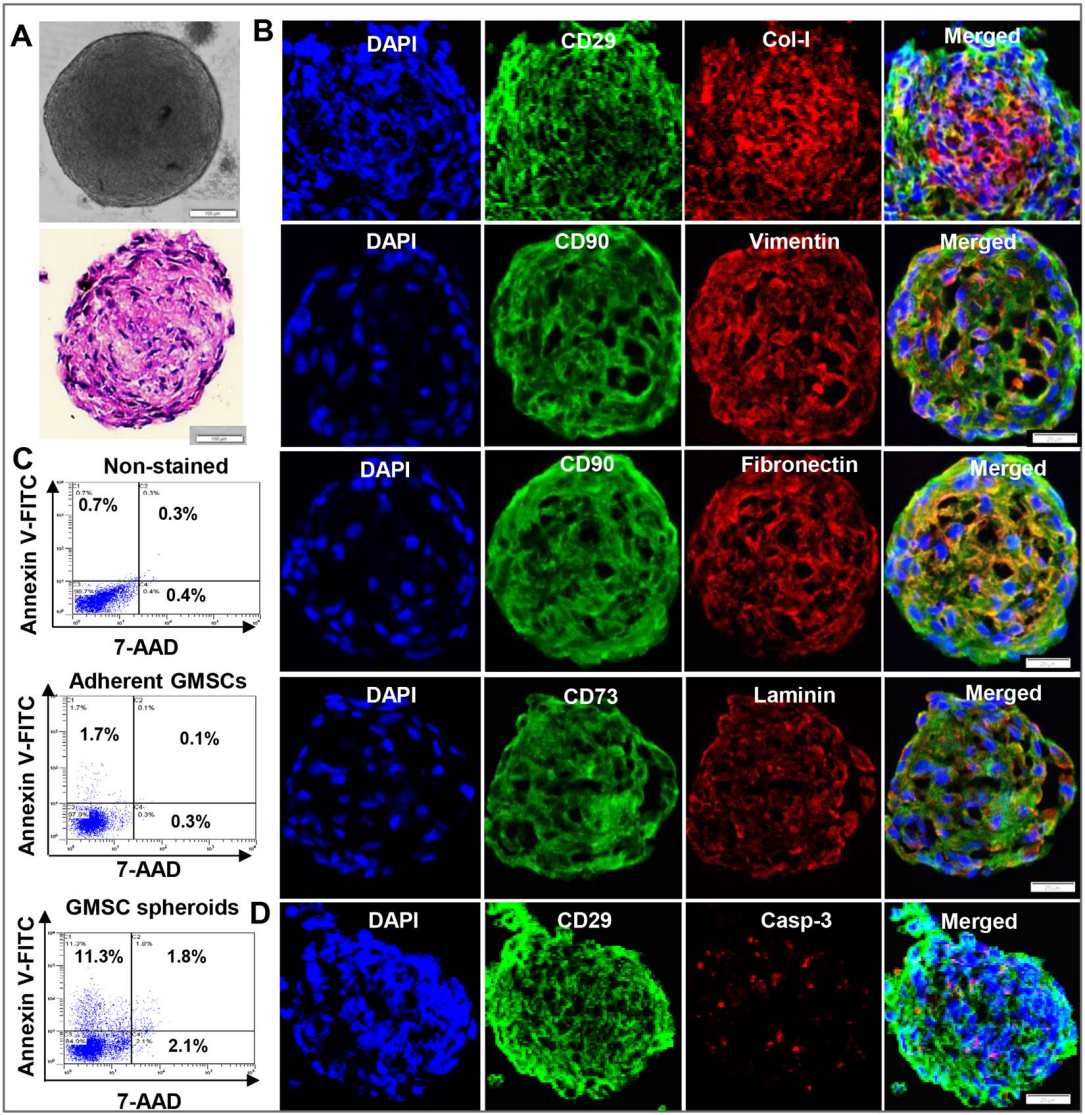
Generation of 3D-spheroid GMSCs. (**A**) 4 × 10^4^ of GMSCs/well in 200 µl of complete stemgro culture medium was seeded into each well of ultra-low attachment round-shaped 96-U well plates and cultured for 48 h. H & E staining of cryosections of GMSC spheroids. Scale bar: 50 µm. (**B**) Immunocytochemistry showed the expression of a panel of MSC-associated markers CD29, CD73 and CD90 and extracellular components such as type I collagen (col-I), vimentin, fibronectin, and laminin in GMSC-derived spheroids. (**C**) GMSC spheroids were dissociated into single cells and stained with Annexin V-FITC and 7-AAD to detect early apoptosis and late apoptotic/necrotic cells by flow cytometric analysis. (**D**) Immunocytochemistry showed that less than 10% of cells inside GMSC spheroids were positive for the cleaved caspase-3. Cell nuclei were counter-stained by DAPI (blue). Scale bar: 50 µm. Data are representative of 3 independent experiments.

We first generated GMSC spheroids by plating a total of 3~4 × 10^4^ of GMSCs/well into the ultra-low attachment round-shaped 96-U well plates (Sumitomo Bakelite, Tokyo, Japan) and cultured with a defined serum-free growth factors supplemented medium (Corning™ stemgro™ hMSC Media) for 48 h_._ The developed round-shaped spheroids were compact with a diameter ranging from 400~500 µm (Fig. 1A). Dual-color immunofluorescence studies showed that GMSC spheroids express a panel of MSC-associated cell surface markers, including CD29, CD73 and CD90 (Fig. 1B). Histological and immunocytochemical studies indicated that these GMSC spheroids were able to produce their own extracellular matrix (ECM), including type I collagen, vimentin, fibronectin and laminin (Fig. 1A,B). We performed dual staining with FITC-Annexin V and 7-AAD and flow cytometric analysis to detect both apoptotic and necrotic cells in GMSC spheroids^18^. Our results showed that about 10% of cells dissociated from GMSC spheroids were early apoptotic cells as characterized by Annexin V(+)/7-AAD(−), while less than 2% of cells were late apoptotic/necrotic cells as characterized by Annexin V(+)/7-AAD(+) (Fig. 1C). Correspondingly, immunofluorescence studies showed that about 10% of cells inside the spheroids were positive for caspase 3, a specific marker for apoptotic cells (Fig. 1D). These findings suggest that GMSCs form compact 3D-spheroids in the size range qualified for 3D bio-printing.

### Neural differentiation of GMSCs under both 2D- and 3D-collagen scaffold conditions

**Figure 2:**
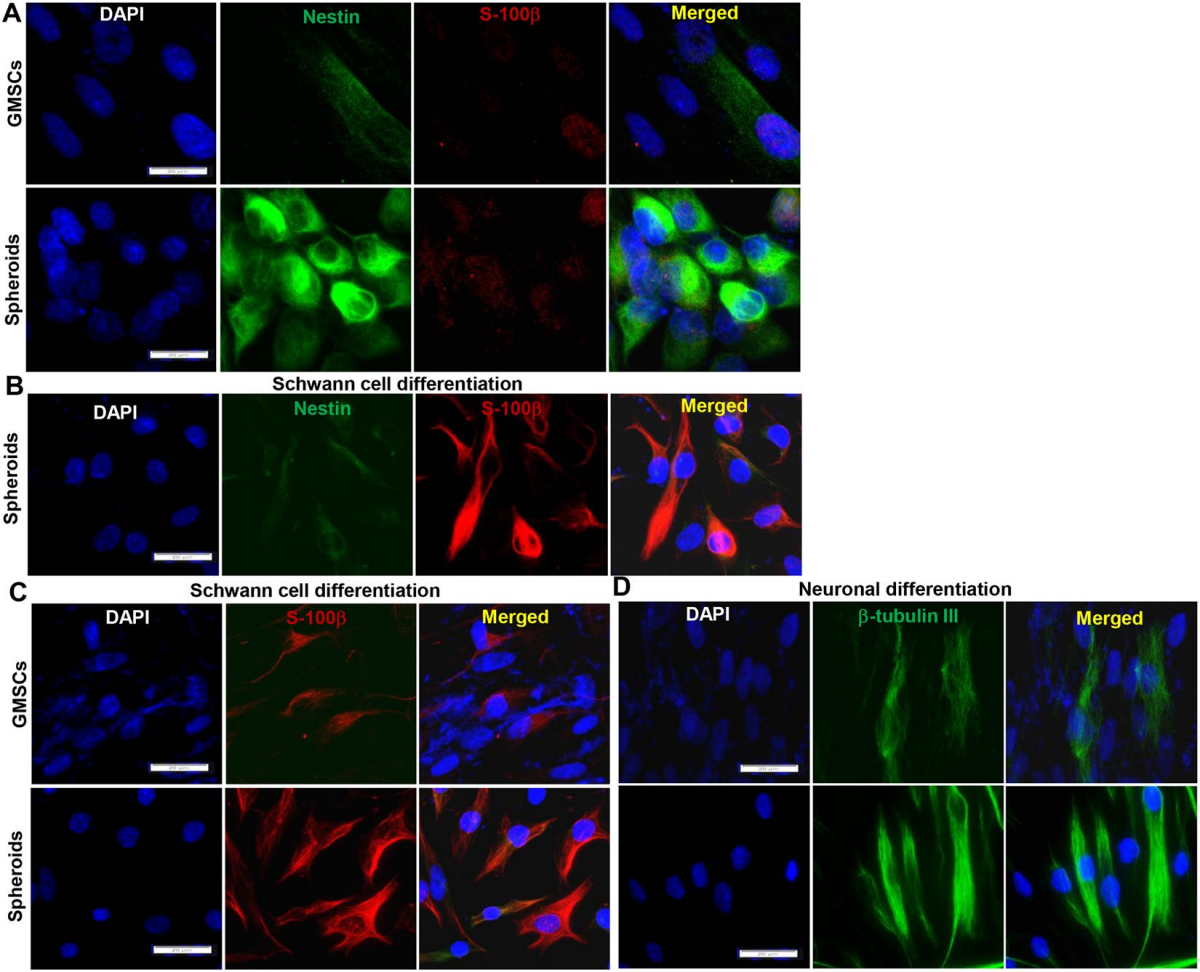
Differentiation of GMSC spheroids into Schwann and neuronal cells under 2D-induction conditions. (**A**) GMSC spheroid cells (spheroids) showed increased expression of Nestin, a neural progenitor cell (NPC) marker, as compared with the adherent counterparts (GMSCs). (**B**) GMSC spheroid cells were seeded onto poly-D-lysine pre-coated 4-well chamber slides and cultured under Schwann cell differentiation conditions for 14 days. The expression of S-100β and Nestin was determined by immunofluorescence studies. (**C**) GMSC spheroid cells (spheroids) and the adherent counterparts (GMSCs) were seeded onto poly-D-lysine pre-coated 4-well chamber slides and cultured under Schwann cell differentiation conditions for 14 days, and the expression of S-100β was determined by immunofluorescence studies. (**D**) GMSC spheroid cells (spheroids) and the adherent counterparts (GMSCs) were seeded onto poly-D-lysine pre-coated 4-well chamber slides and cultured under neuronal cell differentiation conditions for 14 days, and the expression of β-tubulin III was determined by immunofluorescence studies. Cell nuclei were counter-stained by DAPI (blue). Scale bar: 20 µm. Data are representative of 3 independent experiments.

We have recently reported that neural crest-like stem cells (NCSCs) induced from human GMSC spheroids showed increased expression of NPC-related markers, including Nestin, as compared to the adherent counterparts, and GMSC-induced NCSCs exhibited enhanced differentiation potential into both neuronal and Schwann-like cells^19^. Herein, we also observed a significant increase in the expression of Nestin in GMSC spheroid cells cultured under neurosphere-forming conditions as compared with that in the adherent counterparts (Fig. 2A). Under culture conditions for Schwann cell induction for two weeks, most of GMSC spheroid cells lost the expression of Nestin but simultaneously expressed S-100β, a Schwann cell marker (Fig. 2A). In comparison to the adherent counterparts, cells dissociated from GMSC spheroids exhibited enhanced differentiation potential into both Schwann-like cells and neuronal cells (Fig. 2C,D).

**Figure 3:**
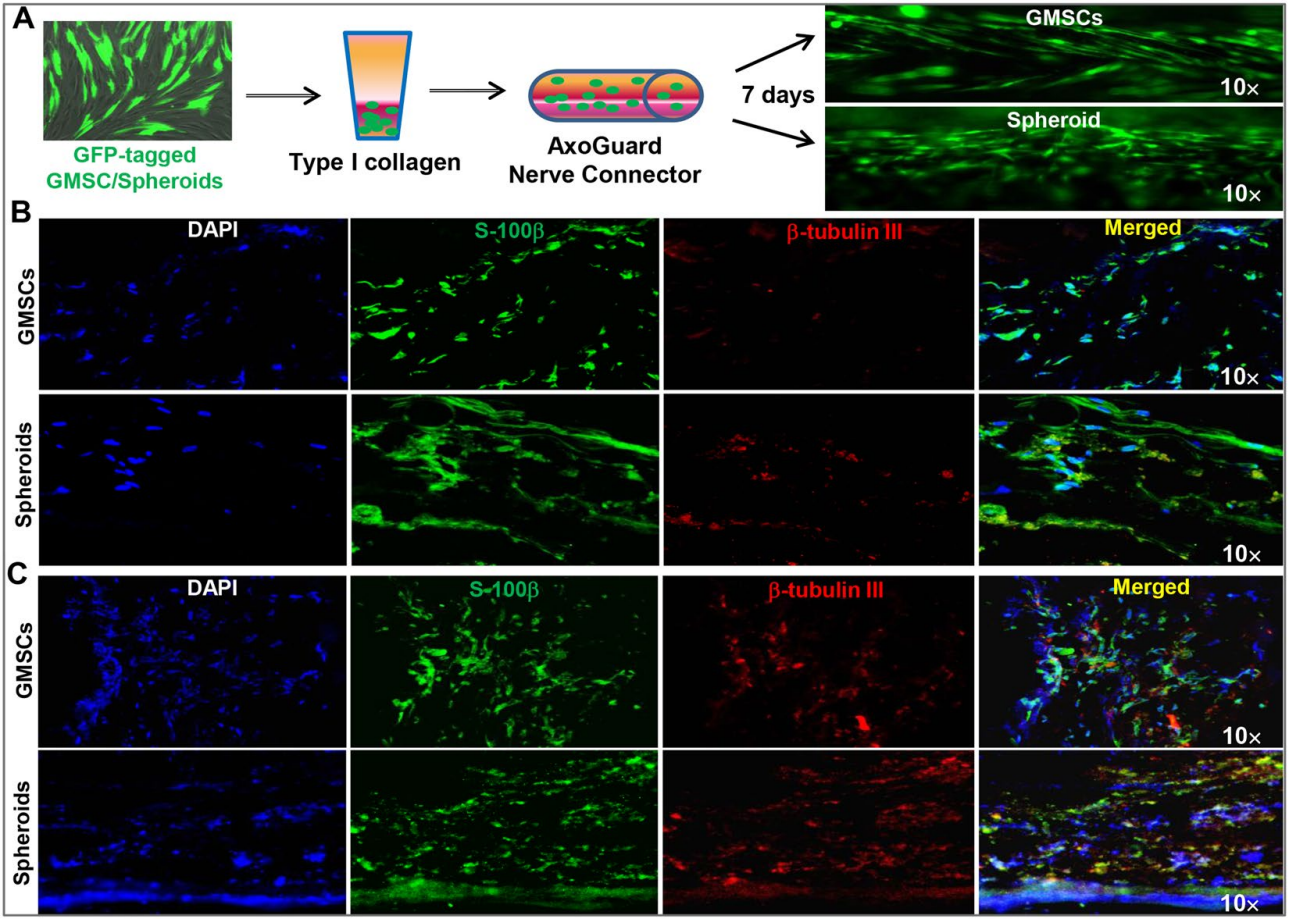
Differentiation of GMSC spheroids into Schwann and neuronal cells under 3D-induction conditions. (**A**) GMSCs or GMSC spheroids stably transduced with a Lentiviral-GFP vector were mixed with bovine type I collagen gel and filled into an Axoguard® nerve protector (3-mm diameter) and cultured in α-MEM containing 10% FBS and antibiotics for 7 days and observed under a fluorescence microscope. (**B**) GMSC spheroid cells (Spheroids) or the adherent counterparts (GMSCs) were mixed with bovine type I collagen gel and filled into an Axoguard® nerve protector (3-mm diameter) and cultured in Schwann cell differentiation medium for 14 days. The structure was fixed in 4% paraformaldehyde and cryosections were cut for immunofluorescence staining of S-100β (green) and β-tubulin III (red). (**C**) GMSC spheroid cells (Spheroids) or the adherent counterparts (GMSCs) were mixed with bovine type I collagen gel and filled into an Axoguard® nerve protector (3-mm diameter) and cultured in neuronal cell differentiation medium for 14 days. The structure was fixed in 4% paraformaldehyde and cryosections were cut for immunofluorescence staining of S-100β (green) and β-tubulin III (red). Cell nuclei were counter-stained by DAPI (blue). Scale bar: 100 µm. Data are representative of 2 independent experiments.

We next determined the multipotent neural differentiation abilities of GMSCs spheroids and the adherent counterparts under 3D-collagen scaffold conditions. GMSCs spheroids were dissociated after culturing under neurosphere-forming conditions for 3 days, mixed with type I collagen gel, filled into an AxoGuard neural conduit (3 mm in diameter) and then cultured for 7 days. Under these conditions, we observed that cells filled in the conduit reorganized and formed aligned structures (Fig. 3A). In addition, under Schwann cell or neuronal induction conditions, GMSC spheroids were found to be more capable of differentiating into S-100β^+^ Schwann-like or β-tubulin III^+^ neuronal cells as compared with their adherent counterparts (Fig. 3B,C). Taken together, these results indicate that GMSC spheroids acquired NPC-like properties characterized by enhanced differentiation into both Schwann and neuronal cells.

### 3D bio-printing scaffold-free nerve tissues from GMSC spheroids

**Figure 4:**
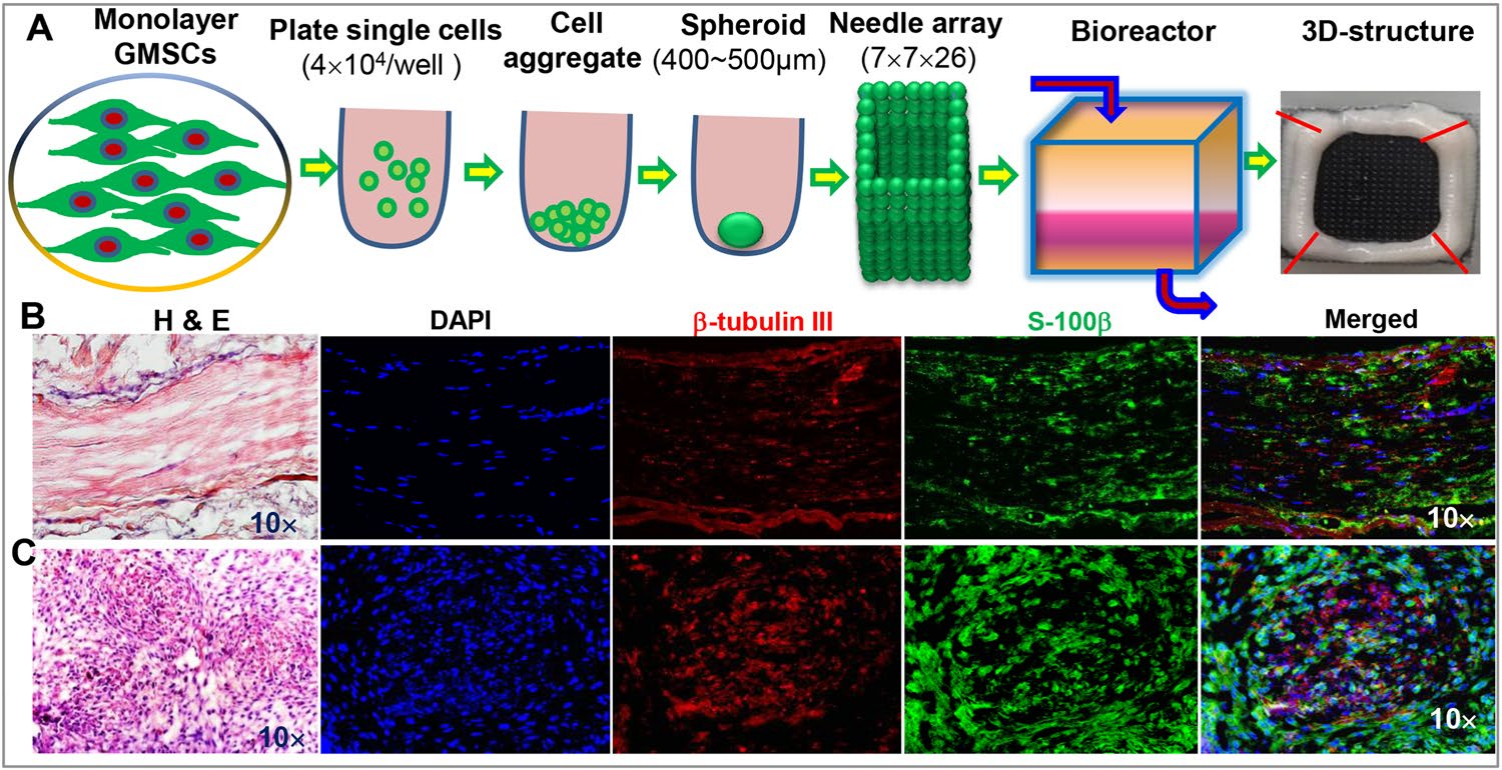
3D bio-printing scaffold-free nerve constructs from GMSC spheroids. (**A**) Procedures for 3D bio-printing scaffold-free nerve constructs from GMSC spheroids. (**B**) Longitudinal section of 3D bio-printed nerve constructs. H&E staining (the left panel); immunofluorescence staining with β-tubulin III and S-100β primary antibodies. (**C**) Transverse section of 3D bio-printed nerve constructs. H&E staining (the left panel); immunofluorescence staining with β-tubulin III and S-100β primary antibodies. Cell nuclei were counter-stained by DAPI (blue). Scale bars: 200 µm. Data are representative of 2 independent experiments.

We next explored the feasibility of 3D bio-printing scaffold-free nerve tissues using uniformly round-shaped spheroids with an average diameter of 400~500 µm that were generated by culturing GMSCs in the ultra-low attachment round-shaped 96-U well plates as described in Fig. 1. Briefly, GMSCs spheroids were collected and assembled into a 9 × 9 mm square needle-array with 3.2 mm in length on each side in the software designed configuration and allowed to fuse into 3D-tubular structures (Fig. 4A). Following further maturation in a bioreactor for 7–10 days, a stable, suturable 3D-nerve construct with a diameter of 2.0 mm was generated (Fig. 4A). Histological analysis of longitudinal and transverse sections of the 3D bio-printed nerve grafts showed aligned nerve-like structures (Fig. 4B,C). Furthermore, immunofluorescence studies revealed the aligned 3D-bioprinted structures that expressed both neuronal marker β-tubulin III and Schwann cell marker S-100β (Fig. 4B,C). These results have demonstrated for the first time the feasibility of 3D bio-printing of scaffold-free nerve tissues from GMSC spheroids.

### Implantation of 3D bio-printed scaffold-free nerve tissues into segmental defects of rat facial nerves

**Figure 5:**
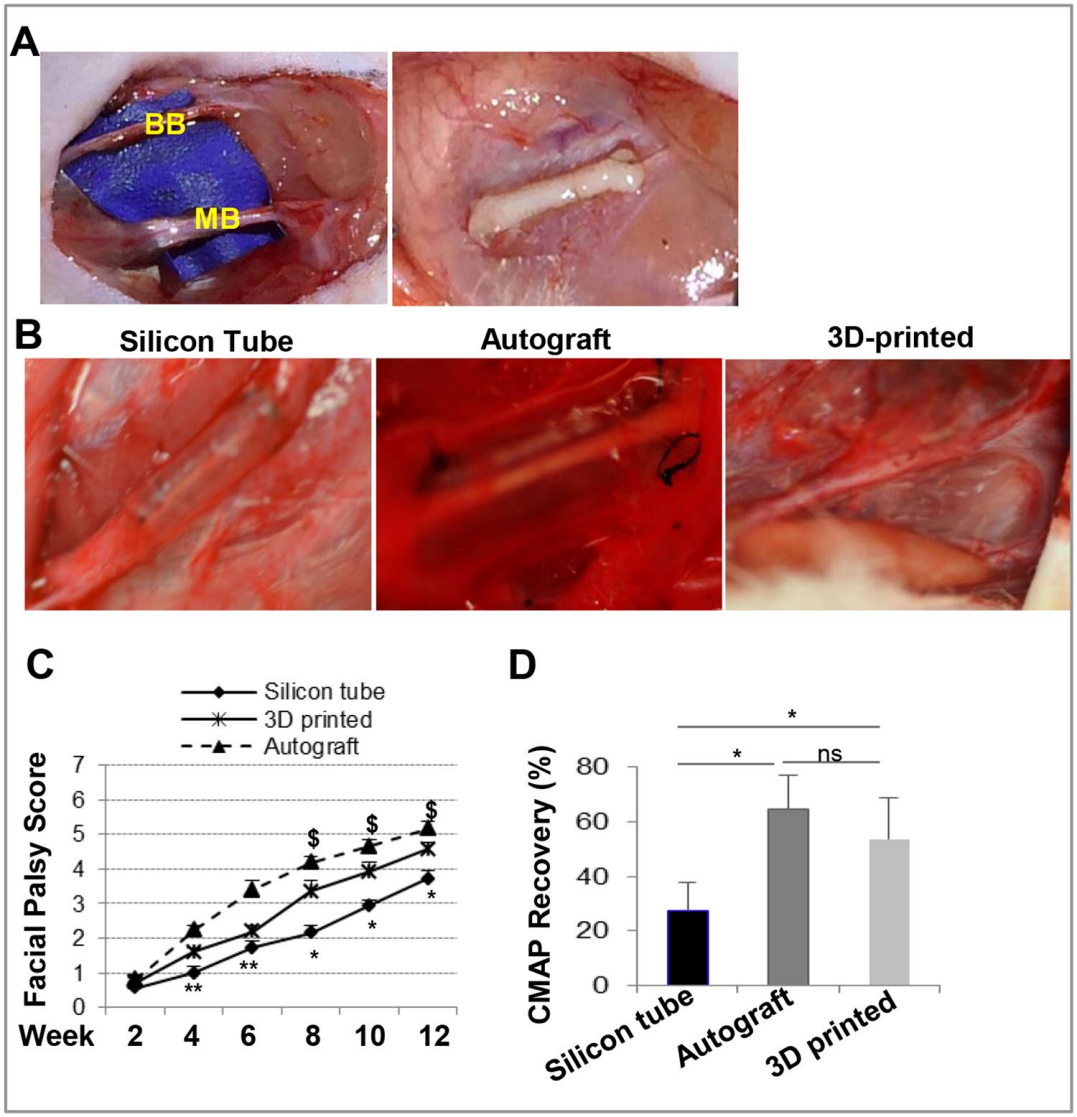
Transplantation of 3D bio-printed scaffold-free nerve tissues from GMSC spheroids promote regeneration of rat facial nerve defects. (**A**) A 5-mm defect was created into the buccal branch of rat facial nerve and bridged with 3D bio-printed nerve constructs. (**B**) Clinical photographs showing the regenerated facial nerve tissues at 12 weeks after transplantation with silicon tube, autograft facial nerve or 3D bio-printed nerve constructs. (**C**) Facial palsy scores evaluated at different time points following transplantation (n = 4). *P<0.05, **P < 0.01, 3D bio-printed grafts *v.s*. silicone tubes; ^$^*P* < 0.05, 3D bio-printed grafts *v.s*. autografts. (**D**) Compound muscle action potential (CMAP) recordings of the vibrissa muscles (n = 3). *P<0.05, **P < 0.01; ns, no significant difference. Data are representative of 2 independent experiments.

We next tested the feasibility of transplantation of 3D bio-printed nerve grafts into segmental defects of the buccal branch (BB) of the facial nerve of rats and observed facial nerve regeneration at 12-week post-transplantation (Fig. 5A). Macroscopically, nerves in control animals using a reverse autograft were covered with normal granulation tissue without neuroma formation. Various levels of nerve remodeling and regeneration were observed in both empty silicon conduit control and 3D bio-printed graft transplantation groups, as the regenerated nerve diameter at the defect segment reached normal size in 3D bio-printed graft transplantation as compared to that from silicon tube control group which did not match the diameter of the native nerve by the terminal time points (Fig. 5B). Clinical outcome assessment of facial nerve recovery was carried out post-transplantation. Functionally, the autograft transplantation group had the best facial palsy score at all time points observed, while the 3D bio-printed graft transplantation group showed higher facial palsy scores than silicon tube control group at 7 weeks after transplantation (Fig. 5C). Following nerve stimulation, compound muscle action potential (CMAP) recordings of the vibrissa muscles indicated that the 3D bio-printed graft transplantation group showed similar CAMP recovery to the autograft transplantation group at 12 weeks after transplantation (Fig. 5D).

### Histological analysis of regenerated rat facial nerves

**Figure 6:**
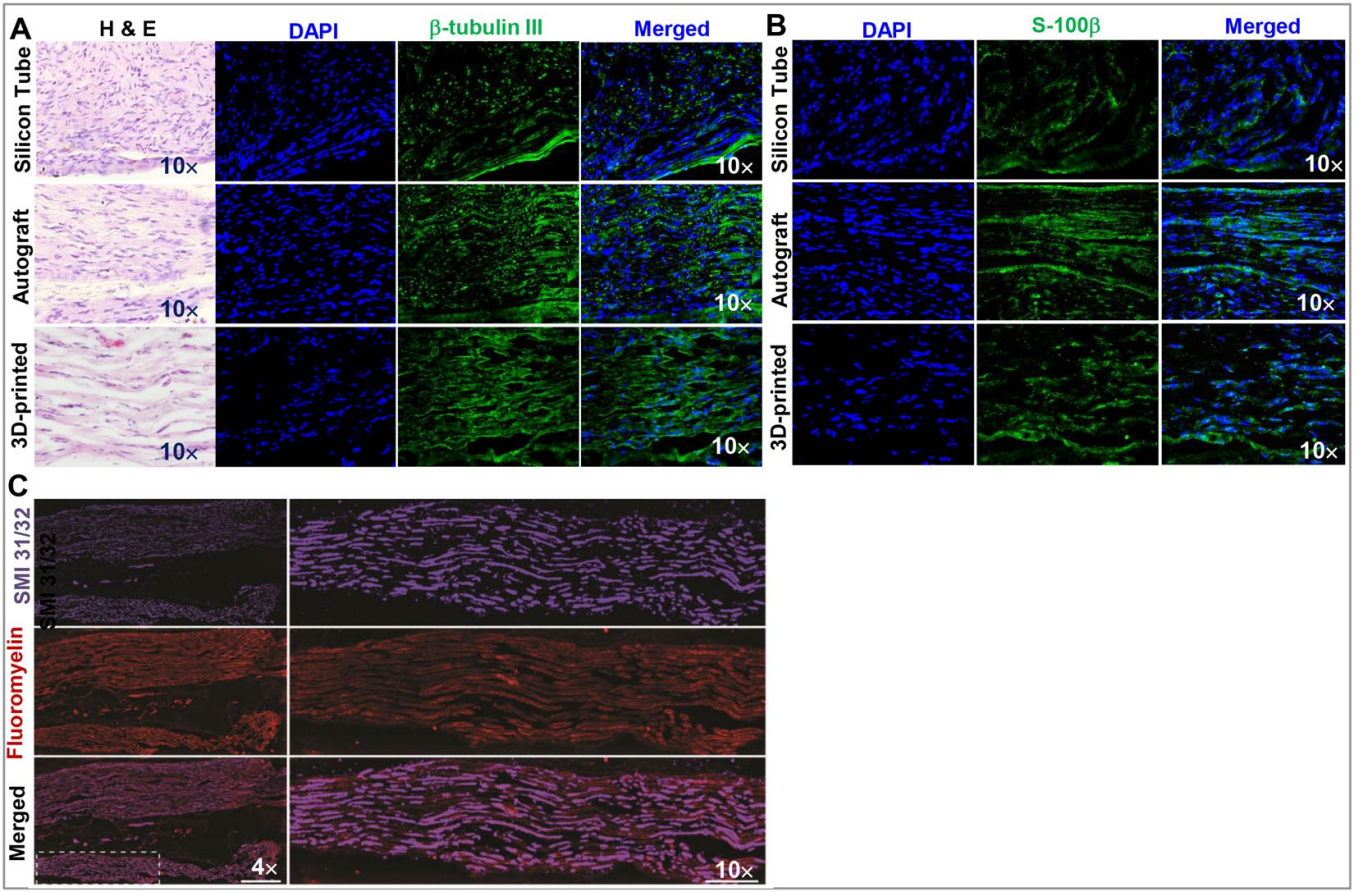
Histological analysis of newly regenerated rat facial nerves. (**A**) Left panels, H & E staining of cryosections of regenerated facial nerves; Immunohistochemistry showed increased expression of β-tubulin III and organized axonal alignment in regenerated nerves from 3D bio-printed construct transplantation as compared with silicon tube transplantation. (**B**) Immunohistochemistry showed increased expression of S-100β in regenerated facial nerves from the group with 3D bio-printed construct transplantation as compared with silicon tube control group. Scale bar: 200 µm. Cell nuclei were counter-stained by DAPI (blue). (**C**) Immunostaining of matured axonal and myelin markers, SMI31/32 and Fluoromyelin, with cryosections of regenerated facial nerves from rats transplanted with 3D bio-printed nerve grafts.

We then performed histological analysis of the regenerated facial nerves by H&E staining as well as immunofluorescence studies on the expression of β-tubulin III and S-100β, a marker of neuronal and Schwann cells, respectively. Our data showed that regenerated nerves from the 3D bio-printed graft transplantation group displayed similarly organized nerve fascicles to those in transplanted autografts; whereas those from silicon tube control group showed disorganized neural fibers (Fig. 6A, the far left panels). Immunofluorescence studies revealed similar expression levels of both β-tubulin III and S-100β and an organized axonal alignment in the regenerated nerves from both groups transplanted with 3D bio-printed grafts and the autografts (Fig. 6A,B); whereas disorganized axonal alignment and lower expression levels of β-tubulin III and S-100β were observed in regenerated nerves from the empty silicon tube control group (Fig. 6A,B). The axonal regeneration of the defected facial nerves after transplantation of 3D bio-printed grafts was further confirmed by the positive immunostaining of axonal and myelin markers, neurofilament H (SMI31/32) and myelin (Fig. 6C). These results suggest that 3D bio-printed scaffold-free nerve grafts may serve as an alternative approach for repair and regeneration of facial nerve defects.

## Discussion

Injuries to peripheral nerves, including facial nerve, usually lead to long-term disability and decreased function of the end target organs^1^. Despite advances in microsurgical techniques and improved management of peripheral nerve injuries, the full functional recovery of the injured facial nerve remains a significant clinical challenge, especially when there is a large gap between the nerve stumps^2,20^. Due to significant disadvantages of utilizing nerve autograft for nerve gap reconstruction^1,2^, much work has been done in the last several decades to develop a variety of synthetic and natural nerve guidance devices to improve peripheral nerve regeneration following injuries with small gaps. However, the overall clinical outcomes of these approaches are still disappointing, as no suitable replacement for autografts has yet been developed^1^.

Most recently, there has been growing enthusiasm in using stem cell-based therapies in combination with tissue engineering, particularly the state-of-the-art 3D bio-printing, for peripheral nerve regeneration^11^. Generally, tissue engineering involves incorporation of biomaterials to provide optimal environmental cues with various types of cells and biologically active molecules. However, use of biomaterials may encounter multiple issues, including limited cell incorporation within a scaffold and scaffold-induced foreign body responses^21^. Despite being in its infancy, 3D bio-printing has been successfully used for generation of several transplantable tissues, including skin, cartilage, bone, liver, vascular constructs, and heart tissues^4,5^. Several studies have also demonstrated the feasibility to fabricate 3D neural tissues using stem cell-laden scaffold as the “bioink”^22,23^. However, due to the limited choices of biomaterials and cellular components as suitable “bioinks” for 3D bio-printing, a platform of scaffold-free fabrication has emerged as a novel approach in tissue engineering^7,8,21,24^. Most recently, a novel 3D bio-printing technique based on the principle of self-assembly/self-organization of tissues has been successfully used to generate scaffold-free transplantable blood vessels using multiple cell spheroids as the building blocks^7^ and 3D nerve conduits developed from human fibroblasts^8^.

One of the major challenges facing the field of stem cell-based tissue engineering, including 3D bio-printing of complex tissues and organs, particularly the neural tissues, is the lack of a readily accessible source of stem cells. Previous studies have demonstrated that numerous adult tissues, including most of the oral and craniofacial tissues, originate from ectoderm-derived migratory neural crest cells during development^25,26^, while specific subpopulations of adult progenitor cells^27^, particularly those from oral and craniofacial tissues^16,28–30^, confer neural crest stem-like cells properties. Most recently, we have shown that GMSCs of neural crest-origin could be easily and readily converted into neural progenitor-like cells and exhibited enhanced capability of differentiating into neuronal and Schwann cells both *in vitro* and *in vivo*^17^. In the current study, we used a novel 3D bio-printing technology and successfully generated scaffold-free nerve constructs using GMSC spheroids as the only cellular component, which exhibited aligned nerve-like structures expressing both neuronal marker β-tubulin III and Schwann cell marker S-100β (Fig. 4). Most importantly, after implantation into segmental defects of rat facial nerves, they displayed comparable beneficial effects to autograft transplantation on the regeneration of defected rat facial nerves (Figs 5 and 6). These findings suggest that GMSCs may represent a promising and easily accessible stem cell source for tissue engineering of nerve tissues.

To date, several types of basal medium have been reported for MSC culture and expansion, including Iscove’s Modified Dulbecco’s Media (IMDM), Dulbecco’s Modified Eagle’s Media (DMEM), and alpha-minimum essential medium (α-MEM), etc.^11^. One of the major hurdles in *ex vivo* expansion of MSCs is the wide use of fetal bovine serum (FBS) in both research and clinical studies, which may cause several inherent problems, such as potential cross-species contamination, undesired xenogenic contents, and batch-to-batch variations^31^. Even though the Food & Drug of Administration (FDA) of USA has approved the use of GMP clinical grade FBS, the immunogenicity against FBS proteins has been reported to reduce the therapeutic efficacy^11,32^. In recent years, much effort has been focused on the development of defined serum-free and nonxenogenic media for the culture and *ex vivo* expansion of MSCs^11,33,34^. In the present study, α-MEM supplemented with 10% FBS was used for *ex vivo* expansion of human GMSCs, while defined serum-free Corning stemgro hMSC Medium and neural basal medium supplemented with growth factors were used for spheroid culture, 3D bio-printing, and maturation of the generated nerve constructs. Future studies are necessary to further define the conditions for large-scale expansion of GMP grade GMSCs for bioengineering of nerve grafts to meet the increasing clinical needs and the manufacture standards for medical device.

In the present study, a scaffold-free nerve graft was 3D bio-printed from human GMSC spheroids, which harbor mechanical properties for surgical handling, suturing, and implanting at the segmental defect sites of rat facial nerves. However, it is noteworthy that we have identified several limitations in our current studies, which need further exploration in future studies. These include (1) further optimizing the time and media used for spheroid generation as well as the fusion and maturation of the 3D bio-printed nerve structures to achieve mechanical properties of the structure (e.g. it may be advantageous for these constructs to better match the stiffness of native nerve); (2) evaluating the functional properties of the 3D bio-printed nerve grafts, e.g. the transmission of nerve impulse in comparison with autologous grafts before and after implantation; (3) demonstrating reproducibility of our findings by increasing the power of the experiments (e.g. the number of animals in each experimental group) and the number of time points evaluated; (4) determining the cell fate and physiological functions of the 3D bio-printed GMSCs *in vivo* following implantation, including assessment of cell viability, proliferation, potentials to differentiate into neuronal and Schwann cells, and/or ability to produce certain types of neurotrophic factors; (5) evaluating various combinations of 3D bio-printed nerve grafts with synthetic or natural nerve conduits, especially to support these constructs for use in repair of even longer facial nerve defects.

In closing, we demonstrated the fabrication of the first 3D bio-printed scaffold-free nerve constructs using spheroids of human GMSCs, a readily available source of autologous stem cells. These novel constructs were then used to bridge segmental facial nerve defects, while recovery of facial palsy score lagged that achieved following autograft repairs, equivalent levels of axonal regeneration (via histology) and target muscle recovery (via CMAP) were attained by 12 weeks post-repair. Of note, this study used the “reverse” autograft as the performance benchmark, which results in a perfect match of the modality (e.g., relative components of sensory and motor axons) and number of fascicles. In clinical settings, a predominantly sensory nerve (e.g. sural) is used to repair a predominantly motor nerve, resulting in diminished autograft performance due to modality and fascicular mis-match; as such the positive control group used in this study would have performed better than expected clinically. Moreover, constructs were bio-printed using human GMSCs and subsequently implanted to repair nerve injuries in rodents absent of immune-suppression. Collectively, these caveats suggest that even better performance could be achieved clinically when using constructs built on using autologous GMSCs in comparison to conventional (sensory) autografts; however, future preclinical and clinical studies will be required to test this assertion. Overall, our current findings have provided evidence that the 3D bio-printed scaffold-free nerve constructs using GMSC spheroids presented promising beneficial effects on regeneration of defected rat facial nerves, thus holding promise for clinical application to promote the repair/regeneration of injured peripheral nerves.

## Materials and Methods

### Animals

Female Sprague-Dawley rats weighing 200–250 g (6–8 weeks old) were obtained from Charles River Laboratories. All animal procedures were approved by the Institutional Animal Care and Use Committee (IACUC) of University of Pennsylvania and performed in accordance with the National Institutes of Health’s Guide for the Care and Use of Laboratory Animals and conformed to the ARRIVE guidelines. Animals were group-housed in polycarbonate cages (two animals per cage) in the animal facilities with controlled temperature (23 ± 2 °C), 40–65% of humidity and a 12-hour light/dark cycle, fed with a standard laboratory diet and allowed *ad libitum* access to drinking water, and acclimatized for at least 1 week prior to the study.

### Cell cultures

Healthy human gingival tissues were obtained as remnants of discarded tissues under the approved Institutional Review Board (IRB) protocol at University of Pennsylvania. All procedures and methods were carried out in accordance with relevant guidelines and regulations. Informed consents were obtained from all participating human subjects for the collection of fresh tissues. Human gingival tissue-derived MSCs (GMSCs) were isolated and characterized as described previously and were routinely sub-cultured with complete culture medium, α-Minimum Essential Medium (α-MEM; Life Technologies, Carlsbad, CA, USA) containing 10% fetal bovine serum (FBS; Zen-Bio, Inc., NC), 100 units/mL penicillin, 100 µg/mL streptomycin, 2 mM L-glutamine, 100 mM non-essential amino acid, and 55 μM 2-mercaptoethanol (2-ME; Life Technologies)^15^. All the cultures were maintained at 37 °C in a humidified incubator with 5% CO_2_. For sphere-forming culture, GMSCs were plated into Ultra-low attachment culture plates with the defined serum-free culture medium: (Corning™ stemgro™ hMSC Media) (ThermoFisher Scientific, Waltham, MA, USA) mixed with the same volume of neural culture medium: neurobasal medium supplemented with 1% N2, 1% B27, 55 μM β-mercaptoethanol (β-ME) (Life Technologies), 20 ng/mL of epidermal growth factor (EGF), 20 ng/mL of basic fibroblast growth factor (bFGF) (Peprotech, Rocky Hill, NJ, USA), 1% penicillin, and 100 µg/ml streptomycin (Life Technologies). After cultured for 3 days, GMSC spheroids were collected and completely dissociated into single cells using StemPro® Accutase® Cell Dissociation Reagent (STEMCELL Technologies Inc, Cambridge, MA, USA) for further studies.

### Immunocytochemical studies

GMSC spheroids were fixed in 4% PFA for 30 min and washed twice with PBS, followed by embedded in O.C.T. for cryosection cutting (5 µm-thickness). Cultured cells fixed with 4% paraformaldehyde (PFA) or cryosections of GMSC spheroids were blocked and permeabilized for 1 h at room temperature in PBS with 2.5% goat serum and 0.5%Triton X‐100, followed by incubation with the following primary antibodies at the appropriate dilution overnight at 4 °C: Nestin (mouse IgG, 1:250) (EMD Millipore, Burlington, MA, USA), CD29 (mouse IgG, 1:250) (BD Bioscience, San Jose, CA, USA), cleaved caspase-3 (Rabbit IgG, 1:250) (EMD Millipore), type I collagen (rabbit IgG, 1:250) (Rockland Biotech, Limerick, PA, USA), CD73(mouse IgG, 1:250) (BD Bioscience), CD90 (mouse IgG, 1:250) (BD Bioscience), vimentin (rabbit IgG, 1:250) (Boster Biological Tech., Pleasanton, CA, USA), fibronectin (rabbit IgG, 1:200 (Sigma, St. Louis, MO, USA), laminin 1 (rabbit IgG, 1:200) (EMD Millipore), β-tubulin III (mouse IgG, 1:200) (BioRad, Hercules, CA, USA), and S-100β (rabbit IgG, 1:250) (Boster Biological Tech). After washing with PBS, cells were incubated with appropriate secondary antibodies at room temperature for 1 h: Alexa Fluor®594 goat anti-rabbit IgG (FITC) (1:250, BioLegend), Alexa Fluor®488 goat anti-mouse IgG (1:250) (BioLegend, San Diego, CA). Isotype-matched control antibodies (BioLegend) were used as negative controls. Nuclei were counterstained with 4′,6-diamidino-2-phenylindole (DAPI). Images were captured using Olympus inverted fluorescence microscope (IX73).

### Flow cytometric measurement of apoptotic and necrotic cells in GMSC spheroids

Apoptosis and necrosis of GMSC spheroids were detected using the Fluorescein isothiocyanate (FITC) Annexin V Apoptosis Detection kit with 7-amino-antinomycin D (7-AAD) (BioLegend) according to the manufacturer’s instructions^18^. Briefly, GMSC spheroids were dissociated with StemPro® Accutase® Cell Dissociation Reagent (STEMCELLS Technologies, Inc.) and the dissociated single cells were resuspended in 100 μl of Annexin V binding buffer with a cell density of about 5 × 10^6^ cells/ml and mixed with 5 μl of FITC-conjugated Annexin V and 7-AAD. Following incubation at room temperature for 15 min in the dark, 400 μl of Annexin V binding buffer was added and then the stained cells were analyzed using a BD FACSCanto^TM^ II flow cytometer (BD Bioscience).

### Neural differentiation of GMSCs and GMSC spheroids under both 2D- and 3D-collagen scaffold conditions

The Schwann cell differentiation medium is composed of α-MEM (Life Technologies) containing 10% FBS, 35 ng/ml all *trans*-retinoic acid (RA), 5 µM forskolin (Sigma), 10 ng/ml basic fibroblast growth factor (bFGF), 5 ng/ml platelet-derived growth factor AA (PDGF-AA), and 200 ng/ml heregulin-β-1 (PeproTech) (PeproTech)^35,36^. Neuronal differentiation medium is composed of neurobasal medium containing 1 x N2 supplement (Life Technologies), 5% FBS serum, 0.5 µM all-*trans*-retinoic acid (Sigma), and 10 ng/mL of brain-derived neurotrophic factor (BDNF) (PeproTech)^37^. GMSCs or GMSC spheroid-dissociated cells were seeded onto poly-D-lysine pre-coated 4-well slide chamber (2 × 10^4^/well) and cultured under Schwann or neuronal cell differentiation conditions at 37 °C and 5% CO_2_ for two weeks with the media changed every 3 days.

For Schwann or neuronal induction under 3D-conditions, sterile lyophilized powder of bovine collagen I was dissolved to a final concentration of 5 mg/ml in sterile 0.2% (v/v) acetic acid (pH 3–4). Cell-containing collagen gels were prepared by suspending cells in a mixture with 60% collagen I (3 mg/ml), 10% 10 x concentrated DMEM, 1.5% 1 N NaOH, and 1 x DMEM was used to fill the final volume to 100%^38,39^. The mixture was then filled into a 3-mm (diameter) AxoGuard® Nerve Protector (Cook Biotech Inc., IN, USA) and allowed to gel at 37 °C for 30 min. The total cell density of all gels was kept constant at 6.0 × 10^5^ cells/ml. Finally, the 3D cell-seeded constructs were cultured under neuronal or Schwann cell induction conditions at 37 °C and 5% CO_2_ for 14 days with the media changed every 3d as described 2D-differentiation.

### 3D bio-printing scaffold-free nerve tissues laden with GMSCs

To generate GMSC spheroids, a total of 4 × 10^4^ of GMSCs in 200 µl of defined serum-free medium (Corning™ stemgro™ hMSC Media) (ThermoFisher Scientific) were plated into each well of ultra-low attachment round-shaped 96-U well plates (Sumitomo Bakelite, Tokyo, Japan) and cultured for 48 hours at 37 °C and 5% CO_2._ The aggregated round-shaped spheroids with a diameter of ~500 µm were used for 3D bio-printing. According to a 3D structure predesigned on a computer system (3 × 3 × 23), a “Bio-3D Printer Regenova with Kenzan Method” (Cyfuse Biomedical K.K.) was used to assemble and skewers spheroids into a 9 × 9 square needle-array with 3.2 mm in length on each side. In this system, the spheroids will be aspirated by a robotically controlled fine suction nozzle (O.D of 0.45 mm and I.D. of 0.23 mm) from the 96-well plates and inserted into the needle-array. A total of 1932 GMSC spheroids was robotically assembled into 7 layers (276/layer) on the needle array. Following placement onto the needle array, spheroids were cultured in flat bottom tube with 25~35 ml Corning stemgro hMSC Medium for 4d and fused into 3D-solid or tubular structures^7^. Afterwards, the generated 3D-structures were put into a Perfuse Chamber filled with 100 ml of Corning stemgro hMSC Medium and 100 ml of neural culture medium (neurobasal medium supplemented with 1%N2, 1% B27, 55 μM β-ME, 20 ng/ml bFGF, 20 ng/ml EGF, 1% penicillin, and 100 µg/ml streptomycin) and collected to a Pump (Terumo, Tokyo, Japan) and allowed for maturation for 10 days in the bioreactor^7^, during which half of the mixed Corning stemgro hMSC Medium and neural culture medium (1:1) was changed every 2 days. Ultimately, solid and tubular 3D-nerve grafts of 1.5 mm in diameter and 8 mm in length were obtained for animal studies. Meanwhile, a 4-mm segment of the 3D bio-printed grafts was fixed in 4% paraformaldehyde and cryosections were prepared for histological and immunohistochemical (IHC) studies.

### Surgical procedures of facial nerve transection

Adult Sprague-Dawley female rats (8 weeks old) were anesthetized intraperitoneally with ketamine/xylazine (100/10 mg/kg). Following sterile surgical technique, a preauricular incision with a marginal mandibular extension was made in the left face of the rat. Buccal and marginal mandibular branches of the facial nerves were exposed and a 5-mm gap was created in the buccal branch and bridged with a 3D bio-printed nerve grafts or autografted nerves using 8–0 Ethilon interrupted sutures. To block the signal to the whisker pad, a 5-mm defect was created in the marginal mandibular branch and ligated with 7–0 nylon sutures. The muscle layers and skin were closed in a layered fashion^40–42^.

### Facial functional analysis using the facial palsy score

Facial palsy scores were blindly evaluated from animals in different treatment groups at every week until the termination of the study according to the standards as described previously^41^. The facial palsy score was valued based on the following functional evaluation: (1) Symmetry of the vibrissae at rest (0, asymmetry; 0.5, slightly; 1, normal); (2) Motion of the vibrissae (0, no motion; 1, minor trembling; 2, effective movement; 3, normal); (3) Symmetry of the nose at rest (0, asymmetry; 0.5, slightly; 1, normal); (4) Motion of the nose (0, asymmetry; 1, slightly; 2, normal). A maximum seven-point indicates a normal midface without facial palsy, while a zero-point indicates complete facial palsy of the midface^41^.

### Compound muscle action potential (CMAP) recordings of the vibrissa muscles

We performed CMAP the regenerated facial nerve (left side) and the contralateral normal nerve (right side), which requires conduction along regenerated axons to stimulate the muscle^41^. Following anesthesia, CMAP amplitude will be recorded and calculated as a difference in voltage between the maximum and baseline CMAP amplitude. CMAP duration will be calculated as the time between 2 points where the baseline is crossed by the rising and declining CMAP curves. CMAP latency will be estimated as the time between the stimulus artifact and the point where the baseline is crossed by rising CMAP curve. CMAP recovery (%) = CMAP of regenerated facial nerve/CMAP of the contralateral nerve.

### Histological and immunohistochemical studies

The GMSCs cultured under sphere-forming or 3D-collagen culture/differentiation conditions, the 3D bio-printed nerve constructs, and the rat facial nerve tissues were collected and fixed in 4% PFA for 24 h and cryoprotected in 10%, 20%, and 30% sucrose and embedded in O.C.T. and 8–10 µm-thick cryostat sections were cut. After blocking and permeabilization in PBS with 3% bovine serum albumin (BSA) and 0.5%Triton X‐100 at room temperature for 1 h, the sections were incubated with primary antibodies for β-tubulin III (1:250; BioRad) or S-100β (1:250; Boster Biological Tech.) overnight at 4 °C, followed by incubation with FITC-conjugated secondary antibodies for 1 h at room temperature. Isotype-matched control antibodies (BioLegend) were used as negative controls. Nuclei were counterstained with DAPI. For immunostaining of matured axonal and myelin markers, SMI31/32 and Fluoromyelin (ThermoFisher Scientific), respectively, cryosections of regenerated facial nerves from rats transplanted with 3D bio-printed nerve grafts were rinsed in 1xPBS and then blocked and permeabilized at room temperature using 0.3% Triton-X100 plus 4% normal horse serum (NHS) for 60 min. Primary antibodies diluted in PBS/4%NHS were applied to the sections and allowed to incubate at 4 °C for 12 h. Sections were stained with SMI31/32 (1:1000) and Fluoromyelin (1:500) and cover-slipped with Fluoromount-G. Images were acquired using a Nikon A1R confocal microscope with NIS Elements (Nikon).

### Statistical analysis

All Statistical analyses were carried out using *SPSS* Statistics version 18.0 (IBM, Inc., Armonk, NY, USA). Direct comparisons between experimental and control groups were analyzed by paired Student’s *t* test. One-way analysis of variance (ANOVA) was employed for multiple comparisons. Post-hoc pairwise comparison between individual groups was performed using the Tukey’s test. All data were expressed as mean ± standard deviation (S.D.). A *P-*value of less than 0.05 was considered statistically significant.
